# Strength estimation of a moving ^125^Iodine source during implantation in brachytherapy: application to linked sources

**DOI:** 10.1093/jrr/rru058

**Published:** 2014-07-01

**Authors:** Kenichi Tanaka, Satoru Endo, Kunihiko Tateoka, Osamu Asanuma, Masakazu Hori, Masaru Takagi, Gerard Bengua, Ken-ichi Kamo, Kaori Sato, Hiromitsu Takeda, Masato Hareyama, Koh-ichi Sakata, Jun Takada

**Affiliations:** 1 Department of Medicine, Graduate School of Sapporo Medical University, South 1, West 16, Chuo-ward, Sapporo Hokkaido 060–8543, Japan; 2 Quantum Energy Applications, Graduate School of Engineering, Hiroshima University, 1-4-1, Kagamiyama, Higashi-Hiroshima, Hiroshima 739–8527, Japan; 3 Division of Radiology and Nuclear Medicine, Sapporo Medical University Hospital, South 1, West 17, Chuo-ward, Sapporo Hokkaido 060–8556, Japan; 4 Hyogo Ion Beam Medical Center, 1-2-1, Kouto, Shingu, Tatsuno, Hyogo 679–5165, Japan; 5 Auckland City Hospital, Park Road, Grafton, Auckland 1142, New Zealand; 6 Teishin-kai Radiation Therapy Institute, 1–6, North 44, East 8, Higashi-ward, Sapporo, Hokkaido 007–0844, Japan

**Keywords:** brachytherapy, ^125^I, source strength, moving source, linked source

## Abstract

This study sought to demonstrate the feasibility of estimating the source strength during implantation in brachytherapy. The requirement for measuring the strengths of the linked sources was investigated. The utilized sources were ^125^I with air kerma strengths of 8.38–8.63 U (μGy m^2^ h^–1^). Measurements were performed with a plastic scintillator (80 mm × 50 mm × 20 mm in thickness). For a source-to-source distance of 10.5 mm and at source speeds of up to 200 mm s^–1^, a counting time of 10 ms and a detector-to-needle distance of 5 mm were found to be the appropriate measurement conditions. The combined standard uncertainty (CSU) with the coverage factor of 1 (*k* = 1) was ∼15% when using a grid to decrease the interference by the neighboring sources. Without the grid, the CSU (*k* = 1) was ∼5%, and an 8% overestimation due to the neighboring sources was found to potentially cause additional uncertainty. In order to improve the accuracy in estimating source strength, it is recommended that the measurment conditions should be optimized by considering the tradeoff between the overestimation due to the neighboring sources and the intensity of the measured value, which influences the random error.

## INTRODUCTION

As a summation of findings from dosimetry research [1–5], the recommended formalism for calculating doses in interstitial low-energy brachytherapy is discussed in detail in the AAPM Task Group 43 Updated Protocol (AAPM-TG43U1) [6]. It requires that the estimated strengths of all implanted sources should be verified by a qualified staff person (e.g. a medical physicist) [7, 8]. Measurements are conducted prior to implantation, and sources with the correct strength are used to ensure that brachytherapy doses are kept at an appropriate level. On the other hand, TG64 recommends that the source strength measurement be performed only for a minimum of 10% of the sources. In other words, the strength of 90% of the sources could potentially not be verified before being used clinically.

As a backup for quality assurance, this study proposes a method for estimating the strength of the source while it is being implanted. With the proposed method, it will also be possible to identify human errors such as accidental replacements of the sources with others having incorrect strength. If the strength of the source actually implanted is found to be lower than what was in the treatment plan, a source may be added so that the dose reaches the desired prescription. On the other hand, if the source strength is higher than what was planned, the dose to the patient can be quickly re-evaluated, and the potential radiation damage to normal tissues can be immediately estimated. If necessary, the dose could then be reduced by increasing the distance between the sources. The estimation method proposed here has previously been suggested for use in loose ^125^I source implants [9, 10]. In the present study, this method has been applied to linked sources.

## MATERIALS AND METHODS

### Principle of the proposed method

The speed of a source varies during brachytherapy implantations because it is moved manually. In the method proposed in this study, the strength of a source moving at an unknown and varying speed is estimated by performing short time measurements. The measurement should be completed while the source is in the region where the efficiency of the detector is constant. This was detailed in a previous work [9]. Additionally, the conditions for the counting time and the distance between the detector and the guiding needle were investigated in order to determine the source strength, regardless of the source speed.

### Sources utilized

Four ^125^I seeds (STM1251, Bard Inc., Murray Hill, NJ, USA) were used in this study. One of them was used in the form of a ‘loose source’ (i.e. single source). The other three sources were linked (with a distance between them of 10.5 mm) and separated by spacers (5.5 mm Standard Source Link, and 5.0 mm Extension Source Link, Bard Inc.). The strength of the loose source was 8.48 U. The linked sources had strengths of 8.38 U for Source #1, 8.43 U for Source #2, and 8.63 U for Source #3. The difference between the strengths was within 2%. Sources #1, #2 and #3 approached the detector in that order. The detector is described in the next subsection.

### Experimental set-up

**Fig. 1.**
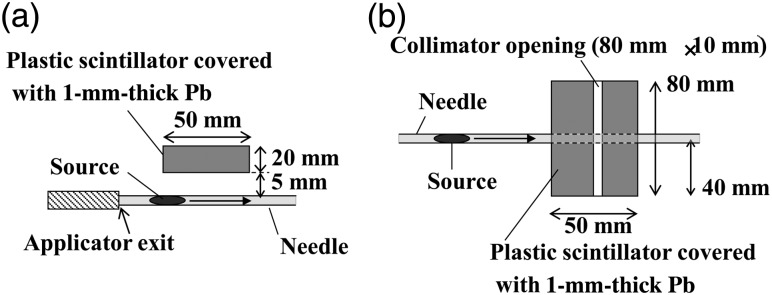
Experimental setup: (**a**) top view; and (**b**) side view. The figures are not to scale.

The experimental set-up is shown in Fig. 1. The source was moved into the needle (#918201, Bard Inc.) by an applicator and a push rod (200-TPV, Mick Radio-Nuclear Instruments Inc., Mount Vernon, NY). The position and speed of the source was controlled with an electric actuator (EZS3D025-A, Oriental Motor Inc., Tokyo, Japan), which was connected to the push rod. In this experiment, the source was not implanted into a patient but only moved in the needle. The detector used was a plastic scintillator (G-tech Inc., Saitama, Japan). This was comprised of the scintillator (EJ200, Eljen Technology Inc., Sweetwater, TX), with dimensions of 80 mm × 50 mm × 20 mm, and the photomultiplier tube (H7416, Hamamatsu Photonics Inc., Shizuoka, Japan).

In order to increase the response of the detector, the detector was set as close as 5 mm from the needle. In addition, the detector-to-needle distance of 20 mm was also tested to investigate the dependence of the detector response on its distance from the source. In order to separate the photons from each of the three linked sources, a collimator was placed on the surface of the scintillator. The collimator was made of Pb and had an opening of 80 mm (width) × 10 mm (length), as shown in Fig. 1b. The collimator was 1-mm thick: enough to shield X-rays from the ^125^I sources. The detector was operated at –1500 V. The signal from the photomultiplier tube was then analyzed with an amplifier (4417, Clear Pulse Inc., Tokyo, Japan) and a single channel analyzer (1150, Clear Pulse Inc.) in turn, and finally counted with a scaler (3340, Clear Pulse Inc.).

In order to reduce the influence of the neighboring sources on linked-source geometry, a grid (MS, Mitaya Inc., Saitama, Japan) was set between the detector and the collimator. One grid, for example, had a density of 3.4 cm^−1^, a grid ratio of 6:1, and a focal distance of 100 cm, as an example. Its focal points were linear, and the line which consisted of the focal points was set perpendicular to the source pathway. The line of the focal points was set vertically, and the source pathway was set horizontally.

### Measurement protocol

At first, the dependence of the detector response on the source position along the needle was measured using the static sources. The static source measurement was performed for both the loose source and the linked sources (i.e. #1, #2 and #3). The measurements were also performed for both geometries, with and without the grid. Measurements of 1-s duration were conducted 10 times.

Following this, the measurement for the moving source was performed for the linked sources only. In this measurement, the reading for the moving source was investigated for its dependence on the counting time. Again, this measurement was performed for both geometries, with and without the grid. The source speed was set at a constant rate of 20, 100 or 200 mm s^–1^. In this case, the measurement was repeated five times at each source speed. The results were compared between the moving and static sources in order to estimate how much the measured value varied as the source speed changed.

In all the measurements in the present paper, a Pb collimator was used, and the detector-to-needle distance was 5 mm. Additionally, for the static source measurement without the grid, the detector-to-needle distance was set at 5 mm, or alternatively at 20 mm.

### Data processing

Among the measured values in each run for the moving source, the maximum was utilized in further analyses, assuming that the source reached the position with the highest detector response when the maximum reading in each run was obtained. In order to investigate the accuracy of the result for the moving source, its ratio against the result for the static source (i.e. moving:static) was computed. This was based on the assumption that the correct result was obtained with the conventional static source measurement. A moving-to-static source ratio close to unity means that the measurement for the moving source was performed correctly.

The uncertainty in the moving-to-static ratio (*Y*) of measured counts was estimated using the method described by Dolan [11, 12]. The procedure is briefly described here. The standard deviation of the moving-to-static ratio among the five runs was regarded as the random (Type A) uncertainty, $\sigma_{Y_{A}}$. Note that the source speed was set at a constant value with the electric actuator in the present study. The impact of the uncertainty in the source speed in actual implantations on the moving-to-static ratio was considered as the systematic (Type B) uncertainty, $\sigma_{Y_{B}}$. The rectangular probability distribution was assumed for a source speed (*s*) up to 200 mm s^–1^. At first, the partial derivative, ∂Y/∂s, was estimated from the following equation:

(1) $$\frac{\partial Y}{\partial s}=\frac{|Y(s_{\max})-Y(s_{\min})|}{s_{\max}-s_{\min}}.$$

Here, *s_max_* is 200 mm s^–1^ and *s_min_* is 0 mm s^–1^, i.e. the static source condition. The standard deviation of the rectangular distribution, $\sigma_{s}$, was computed as

(2) $$\sigma_{s}=\frac{|s_{\max}-s_{\min}|}{2\sqrt{3}}.$$

Then, Type B uncertainty in *Y* due to the source speed uncertainty, $\sigma_{Y_{B}}$, was estimated as

(3) $$\sigma_{Y_{B}}=\frac{\partial Y}{\partial s}\sigma_{s}.$$

Finally, the combined standard uncertainty (CSU) at a convergence factor, *k* = 1, ($\sigma_{Y}$) was estimated as

(4) $$\sigma_{Y}=\sqrt{\sigma_{Y_{A}}^{2}+\sigma_{Y_{B}}^{2}}.$$

## RESULTS

### Measurement for static source

**Fig. 2.**
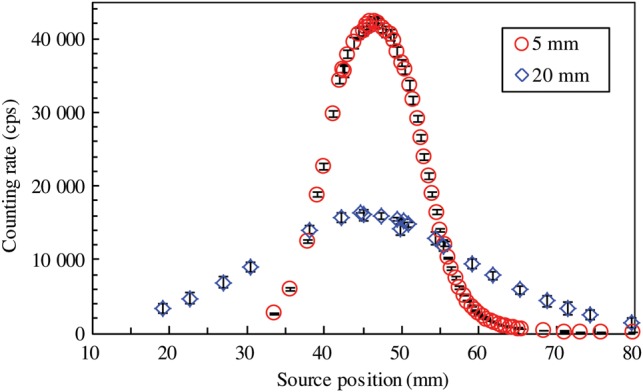
Dependence of detector response on detector-to-needle distance for the static loose source without a grid.

The dependence of the detector response on distance for the static loose source is shown in Fig. 2. The source position is an arbitrary value, which approximately corresponds to the distance from the end of the applicator exit to the source in Fig. 1a. The error bars indicate the standard deviation for 10 measurements. The counting rate of the background signal was 22 ± 2 cps, and this was subtracted from the measured response for the static source shown in Fig. 2. The reading for the detector-to-needle distance of 5 mm reached a maximum of 42 309 ± 724 cps at 46.5 mm. For the distance of 20 mm, the maximum was 16 314 ± 336 cps. Further measurements were performed for the distance of 5 mm to obtain higher counting rates with better statistics.

**Fig. 3.**
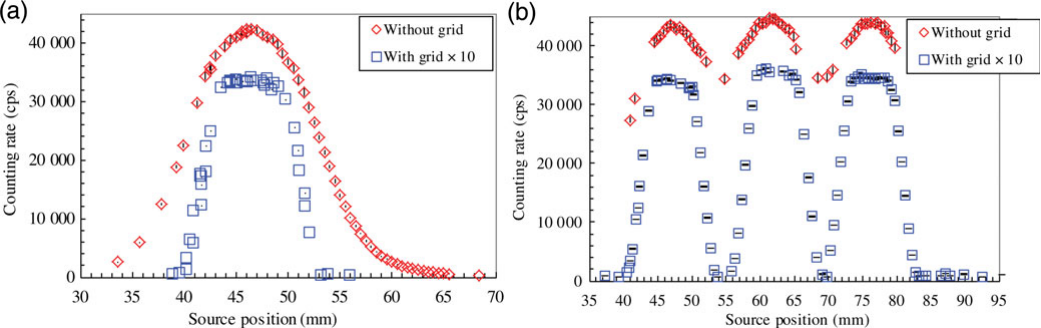
Detector response to static source: (**a**) loose source; and (**b**) linked sources at a source-to-source distance of 10.5 mm.

The detector response measured for the static source is also shown in Fig. 3. The source position for the linked sources in Fig. 3b is approximately the same as the distance from the applicator exit to Source #1. For the measurements without the grid, the response for the linked sources had peaks of 43 547 ± 789 cps at 46.8 mm for Source #1, 44 666 ± 721 cps at 61.3 mm for Source #2, and 42 889 ± 770 cps at 74.3 mm for Source #3. The interval between the three peaks is ∼15 mm, which is the same as the distance between the source centers. This distance is the sum of the length of the spacers (10.5 mm) and the source (4.5 mm). The contribution to the reading by the neighboring sources was estimated from the distribution for the loose source in Fig. 3a. The reading at 15 mm away from the maximum was 1639 ± 55 cps at 61.5 mm. This corresponds to ∼4% of the maximum. Accordingly, assuming all the sources have the same strengths, the reading for the linked source will be overestimated by ∼4% when there is one neighboring source, and ∼8% when there are two neighboring sources. When the influence of more distant sources was considered, the reading at ∼30 mm away from the maximum was ∼0.3%, 145 ± 12 cps at 75.9 mm. This is much smaller than those obtained at 15 mm away. This overestimation is expected to contribute to the uncertainty in the proposed source strength measurement.

The results of the measurements where a grid was used are also shown in Fig. 3. For clarity, the data are plotted after being multiplied by a factor of 10. In this case, the distribution has a sharper increase, flatter plateau, and sharper decrease in both Fig. 3a and b. For the loose source, the maximum was 3416 ± 76 cps at 46.5 mm. The plateau was at ∼44–49 mm, and the response fluctuation was ∼6%. The plateau size of ∼5 mm corresponds to the distance in which a 4.5-mm-long source can move inside the collimator opening of 10 mm. The reading at ∼15 mm away from its maximum was no more than 3 ± 4 cps. The advantage of using the grid is that it minimizes the interference from neighboring sources. However, it decreases the reading by one order.

The fluctuation in the reading in the plateau region, where the source could move inside the collimator opening, was ∼6–7% in both the geometries with and without the grid. In the proposed estimation method, this region is a potential location for the moving source during the measurement. This fluctuation in the reading will create uncertainty in the calculated strength. In actual treatments, it is unknown when the source enters this region. Accordingly, it is practical to perform two or three sets of measurements while the source passes through this region. In this case, one or two of those sets will be completed while the source is inside the region, but part of the others will be performed when the source is outside the region. As an example, 2 mm was tested as the distance that the source travelled during the measurement. We tested the counting times of 100, 20 and 10 ms, at source speeds of 20, 100 and 200 m s^−1^, respectively.

### Moving source measurement

**Fig. 4.**
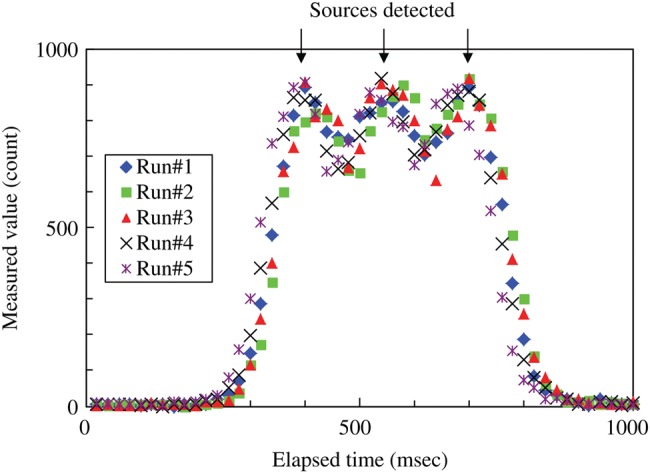
Raw data measured without a grid for a counting time of 20 ms, for linked sources at a source-to-source distance of 10.5 mm moving at 100 mm s^−1^.

**Fig. 5.**
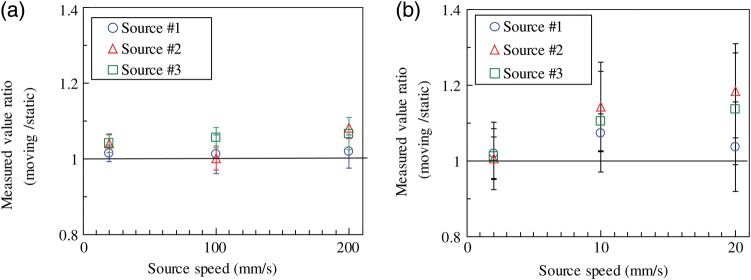
Measured moving-to-static source ratio for linked sources: (**a**) without a grid; and (**b**) with a grid. Three sources were linked with a distance between them of 10.5 mm. The counting time was set at 100 ms for the source speed of 20 mm s^−1^, 20 ms for 100 mm s^–1^, and 10 ms for 200 mm s^−1^, so that the source advanced by 2 mm during a measurement.

An example of the raw data is shown in Fig. 4 for the source moving at 100 mm s^–1^ with the counting time of 20 ms in the geometry without the grid. The measurements for the moving sources were performed as five runs. The average and standard deviation of the maximum in each of the five runs are indicated in Table 1. The ratio against the result for the static source (i.e. moving:static) is shown in Fig. 5. The results of the moving source showed better agreement with those of the static source when the grid was not used. Assuming that the source speed in the implantation ranged up to 200 mm s^–1^, a counting time of 10 ms was applied. In this case, the counting rate in Fig. 3b was converted to the counting time of 10 ms for the static source. As shown in Fig. 5b, the moving-to-static ratio in 10 ms counting time at 200 mm s^–1^ was between 1.02 and 1.08 for the three sources.

**Table 1.** Measured counts for a moving source

	Without grid			With grid		
Source speed (Counting time)	Source #1	Source #2	Source #3	Source #1	Source #2	Source #3
20 mm s^−1^ (100 ms)	4278 ± 50	4500 ± 77	4324 ± 55	329 ± 21	342 ± 17	336 ± 28
100 mm s^−1^ (20 ms)	874 ± 41	889 ± 24	899 ± 17	71 ± 3	80 ± 8	75 ± 9
200 mm s^−1^ (10 ms)	435 ± 17	455 ± 8	449 ± 16	34 ± 4	41 ± 4	38 ± 5

The uncertainty in the moving-to-static ratio of measured counts obtained for the 10 ms counting at the speed of 200 mm s^–1^ was estimated. The results are listed in Table 2. The Type B uncertainty ranged from 0.5–2.2%. The CSU (*k* = 1) for the measurements without the grid was 3.5–4.7%. The aforementioned overestimation due to the neighboring sources was compensated for by performing both the static and the moving source measurements using the same linked sources. However, compensation is not always complete in actual treatments. The reason for this is that the static linked source measurement is conducted using several sources with the same correct strengths, from which the calibration factor to convert the measured counts to source strength (U) will be determined. When some of the moving sources have incorrect strengths in actual implantation, the relationship of the strengths between the sources will be different from that in the calibration, i.e. measurements with static sources. In this case, the calibration factor is no longer appropriate. Therefore, the influence of the neighboring sources at ∼8% should be regarded as a potential source of additional uncertainty, apart from the CSU (*k* = 1) in Table 2.

**Table 2.** Uncertainty estimated for 10 ms counting for source speed of 200 mm s^−1^

	Without grid			With grid		
Components	Source #1	Source #2	Source #3	Source #1	Source #2	Source #3
Type A: Fluctuation of the measured moving-to-static source ratio among five runs (%)	4.3	2.5	4.1	11.4	10.5	12.9
Type B: Error due to unknown source speed (%)	0.5	2.2	1.8	1.0	4.9	3.7
Combined standard uncertainty (*k* = 1) (%)	4.4	3.5	4.7	11.9	13.3	15.1

The counts obtained for measurements using the grid were one order lower, as shown in Fig. 3b. This was reflected in the Type A uncertainty, listed in Table 2, which was as high as 10.5–12.9%. The moving-to-static source ratio for measurements with and without the grid were significantly different, as shown in Fig. 5. The potential uncertainty due to the interference from the neighboring sources was negligible in this case. The grid decreases the contribution from the neighboring sources and helps to distinguish the sources. However, it also reduces the measured signal and increases the Type A uncertainty. Optimization of the conditions applied when using a grid may improve its efficacy.

## DISCUSSION

At a source-to-source distance of 10.5 mm, a condition potentially applicable to the treatment was found for source speeds up to 200 mm s^–1^. The measurement condition corresponded to a counting time of 10 ms and a detector-to-needle distance of 5 mm. The CSU (*k* = 1) was up to 15% when using a grid, and up to 5% without a grid. In the latter, the 8% overestimation due to the neighboring sources potentially contributes to additional uncertainty.

The measurement conditions in this study will also work for source-to-source distances >10.5 mm, e.g. 15.5 mm or greater. In this case, longer counting times are also applicable, which will then reduce the Type A uncertainty and improve the accuracy of the strength estimation.

For a source-to-source distance of 5.5 mm, the length of the collimator opening should be 5.5 mm or less in order to avoid a situation in which two sources are inside the collimator opening simultaneously. In that case, the source can move by increments of 1 mm inside the collimator opening. Because it is unknown when the source comes up to the collimator opening, an example of the counting time is the time taken to move the source by 0.5 mm. In that scenario, the counting time and measured value will be one-fourth of those obtained in the present study. Also, the Type A uncertainty will be doubled. Furthermore, the additional uncertainty due to the two neighboring sources will be as high as 40% when the grid is not used. This is seen in Fig. 3a, in which the counting rate at 10 mm away from its maximum was 8735 ± 166 cps for the loose source with a strength of 8.48 U. This estimation is for the condition where the two neighboring sources have the same strength as the source to be measured. If they have larger strengths, the additional uncertainty due to the neighboring sources is even larger. The usage of the grid may reduce the neighboring source interference, in spite of the lower counting rate leading to higher Type A uncertainty. In applying the proposed method to a source-to-source distance of 5.5 mm, optimization of the detector and grid conditions is recommended.

For a source-to-source distance of 0.5 mm, the length of the collimator opening should be set to as little as 0.5 mm in order to avoid the situation in which two sources are inside the collimator opening. In this geometry, the collimator opening cannot include the entire source. The X-rays from only small sections of the source can be detected, which is not appropriate for the source strength measurement. The application of the proposed method to a source-to-source distance of 0.5 mm is not appropriate.

In actual clinical application, there are many possible positions at which to set the needles. In general, the needles are located within an area of 60 mm × 60 mm. This range influences the detector-to-needle distance, and its impact on the measured data is shown in Fig. 2. For a detector-to-needle distance of 20 mm, without the grid the reading at 15 mm from its maximum was 8192 ± 81 cps for the loose source with a strength of 8.48 U. This is ∼60% of the maximum. The potential overestimation due to the two neighboring sources is as high as 120%, which would mean that the strength of the source will be estimated incorrectly due to the X-rays from the neighboring sources. Again, this estimation is for the condition where two neighboring sources have the same strength as the source to be measured. If they have larger strengths, the additional uncertainty due to the neighboring sources also becomes larger. Furthermore, the linked sources may not even be separated using the temporal change in the measured values shown in Fig. 4. On the other hand, the use of a grid would decrease the influence of the neighboring sources drastically. However, a longer detector-to-needle distance would make the measured value smaller and the Type A uncertainty larger. These disadvantages can be avoided by using a jig when it is required to change the position of the detector, so that the detector-to-needle distance is always set constant for every needle position.

## CONCLUSION

The present paper has proposed a method for estimating the strengths of linked sources during implantation in brachytherapy, regardless of the source speed. For a source-to-source distance of 10.5 mm and at source speeds of up to 200 mm s^–1^, a counting time of 10 ms and a detector-to-needle distance of 5 mm were found to be the appropriate measurement conditions. The CSU (*k* = 1) was up to 15% when using a grid during the measurement, and up to 5% without the grid. In the latter, an 8% overestimation due to the neighboring sources could be a potential source of additional uncertainty. The overestimation can be compensated for to some extent by doing a calibration using linked sources.

The detector should be arranged close to the needle guiding the sources, e.g. at 5 mm away. Accordingly, the jig to arrange the detector should have a function to keep the detector-to-needle distance small by changing the detector position according to the varied needle positions during the implantation. With linked sources, measurement conditions (including the use of a grid, limitation in the counting time because of the collimator, and the necessity for a jig) should be carefully considered. The advantage of using a grid is seen in the reduction of the neighboring source interference. On the other hand, using a grid leads to an increase in the Type A uncertainty. Optimization, especially in the tradeoff between the neighboring source interference and the Type A uncertainty, would improve accuracy in estimating the source strength.

## FUNDING

Part of the present study was supported by a Grant-in-Aid for Scientific Research from the Japan Society for the Promotion of Science (Grant #21791203), and by the Japan Science and Technology Agency under Grants #AS221Z02563F and # HWY2012-1-314. Funding to pay the Open Access publication charges for this article was provided by Sapporo Medical University.
